# Assessing the Formal Integration of a Student-Run Free Clinic for People Experiencing Homelessness Into a Medical School Curriculum

**DOI:** 10.7759/cureus.98335

**Published:** 2025-12-02

**Authors:** Felicia Zhang, Austin Burrows, Corinne Allas, Sarah Goldgar

**Affiliations:** 1 Department of Medicine, David Geffen School of Medicine, University of California, Los Angeles, Los Angeles, USA; 2 Department of Medicine, Division of General Internal Medicine, University of California, Los Angeles, Los Angeles, USA

**Keywords:** clinical skills education, free clinic volunteering, homeless healthcare, homeless patients, medical education curriculum, medical school curriculum, street medicine, student-run free clinic, unhoused

## Abstract

Background

Student-run free clinics (SRFCs) are present in many medical schools and help bridge gaps in healthcare access for people experiencing homelessness. Volunteering for SRFCs is a common medical school experience, though it is seldom an integrated component of medical school curricula. This study aims to assess the benefit of formal integration of an SRFC into a medical school curriculum by investigating the common street-side clinic diagnoses and assessing whether clinic participation improved students’ confidence in their clinical skills and navigation of the social determinants of health (SDHs) and trauma-informed care.

Methodology

A retrospective review of medical charts from 2020 to 2024 was completed to understand the common diagnoses seen at the clinic. First-year medical students enrolled in the SRFC as a part of their first-year curriculum from 2022 to 2024 were given pre-tests and post-tests to assess change in confidence across several clinical skills after participation throughout their first year. The results were analyzed by categorization and using Fisher’s exact test. Qualitative responses to assess their experience were also collected in the post-test and reviewed for systematic themes.

Results

The retrospective chart review showed the most common diagnoses were musculoskeletal pain, viral upper respiratory infection, and cellulitis. It also showed that a variety of conditions were seen across many different organ systems. There was significant improvement for all survey questionnaire items, including clinical skills, inter-team collaboration, trauma-informed care utilization, and understanding the SDHs. Three overarching themes reflected students’ experiences after the fact, including preparation for clinical rotations with real-life applications, independence as a medical student within a safe learning space, and exposure to street-side medicine and humanism.

Conclusions

SRFCs play an important role in healthcare delivery to underserved communities and provide numerous benefits to their student participants. Despite the benefits, there is a lack of exposure for medical students as part of formal medical school curricula. This study shows the benefits of SRFCs with improvement in medical students’ clinical skills, subjective readiness for clinical rotations, and ability to integrate whole-person healthcare. Beyond this, it allows students to foster relationships with a vulnerable patient population and develop skills to bring forward in their careers when working with underserved communities, including increased understanding of the SDHs and trauma-informed care. Although limitations exist within this study, further exploration of the unique nature and impact of SRFC integration into medical school curricula is warranted.

## Introduction

People experiencing homelessness (PEH) face significant barriers to accessing medical services [1]. Student-run free clinics (SRFCs) provide PEH with direct access to low-barrier, accessible care while creating valuable training opportunities for medical students and bridging the gap between classroom learning and clinical practice [2-5]. Literature has shown that SRFCs play an important role in providing preventative care services and chronic disease management [2]. Additionally, estimates have suggested that SRFCs have a positive economic impact, reducing urgent care visits and saving money for healthcare systems [2]. As of the most recently published data in 2014, up to 75% of U.S. medical schools have an SRFC [3]. Students frequently participate in SRFCs as part of medical school, but typically as an extracurricular activity, not through the formal medical school curricula [3]. Prior studies have shown that medical students found their participation in SRFCs provided vital community service and expanded their understanding of the social determinants of health (SDHs) [5]. One recent study assessed how these clinics affect students’ acquisition of clinical knowledge and skills in older medical student learners [4]. To date, none have looked at the integration of an SRFC as a formal part of the medical school curriculum.

The objective of this study was to assess the utility of integrating SRFCs into formal medical school curricula by looking at the common diagnoses seen at the clinic and assessing whether participation in the clinic improved students’ comfort with clinical skills and their ability to navigate SDHs while caring for unhoused patients.

## Materials and methods

Study setting

The Mobile Clinic Project (MCP) at the David Geffen School of Medicine (DGSOM) at the University of California, Los Angeles (UCLA) is a student-run street-side clinic held six times per month in the Hollywood neighborhood of Los Angeles, California. The clinic provides free urgent-care services for patients experiencing homelessness and works to tie patients into the existing continuum of healthcare. The clinic is staffed by UCLA medical students, who see patients under the supervision of attending and resident physicians. Medical students had traditionally been volunteers, participating in their own free time outside the formal medical school curriculum and without receiving school credit.

In the fall of 2021, a new mandatory course called the Early Authentic Clinical Experience (EACE) started at DGSOM for first-year medical students. This course was designed to offer students hands-on experience with clinical skills early on in medical school. First-year medical students were assigned to numerous UCLA-affiliated community and hospital sites across Los Angeles and participated in 12 half-day clinical visits over the course of their first year of medical school. An annual cohort of 15 first-year students chose to complete their EACE course experience at MCP.

At each clinic visit, these medical students were assigned one to two patients and tasked to take a medical history, perform a targeted physical examination, give an oral presentation to the supervising physician, assist with counseling the patient and providing treatments, and document a written note for the encounter. A medical history-taking form was developed to assist our early learners in guiding them through a complete medical history and the documentation process (Appendix A). Students also collaborated with undergraduate caseworkers and public health graduate students who assisted with social service needs.

Data collection

A retrospective chart review of 766 medical visits from 407 unique individuals seen between December 2020 and March 2024 was conducted to assess the medical conditions seen at our clinic and to provide an understanding of the scope of conditions seen by our students. Diagnoses noted in the assessment and plan were grouped by relevant body system (e.g., cardiovascular, musculoskeletal, etc.).

To assess the first-year students’ clinical skill acquisition, a survey was developed and given to two cohorts of first-year medical students (n = 30) participating at MCP for EACE between September 2022 and August 2024. Consent was given before survey administration. Students were given a pre-test survey before their first clinic visit and a post-test survey after their 12th and final visit. Both surveys asked about student comfort with various clinical skills, including taking vital signs, obtaining a medical history, performing physical examinations, summarizing an oral presentation, and generating an assessment and plan. They also asked about comfort with skills needed to navigate health barriers for PEH, including interacting with the interdisciplinary team, using trauma-informed care, providing referrals to primary care, and understanding the SDHs and how they affect our patients. Responses were given on a Likert scale with numerical ratings of 1-4. (4: I can perform independently with minimal, passive supervision; 3: I can perform but need active attending support; 2: I need significant guidance and support from the attending; 1: This is an area of concern for me, and I would like to speak with a faculty member.) The post-survey also included open-ended questions intended for students to reflect on their experience, provide feedback, and comment on their readiness for clinical rotations (see Appendix B for the pre- and post-survey questions and Appendix C for the additional post-survey open-ended questions). The surveys were not validated. The study was approved by the UCLA IRB (IRB#22-001399 and 24-6309).

Statistical analysis

Responses to the pre- and post-survey questions were assessed by grouping Likert responses 1-3 into one category (still needing active guidance and supervision) and comparing them to students who chose response 4 (only needing minimal, passive supervision), presuming this level of confidence was appropriate for students before starting clinical rotations. These two categories were compared using Fisher’s exact test. A significant test was noted as an ɑ <0.0063. This stricter significance level was adjusted to avoid inflating the potential for a Type I error. As the surveys were completed anonymously, the pre- and post-surveys were not matched. Post-test qualitative reflections and feedback were reviewed and assessed for systematic themes. These themes were identified by manually reviewing the raw data and generating an inductive code.

## Results

Quantitative findings

Clinical Conditions Seen at the Clinic

**Figure 1:**
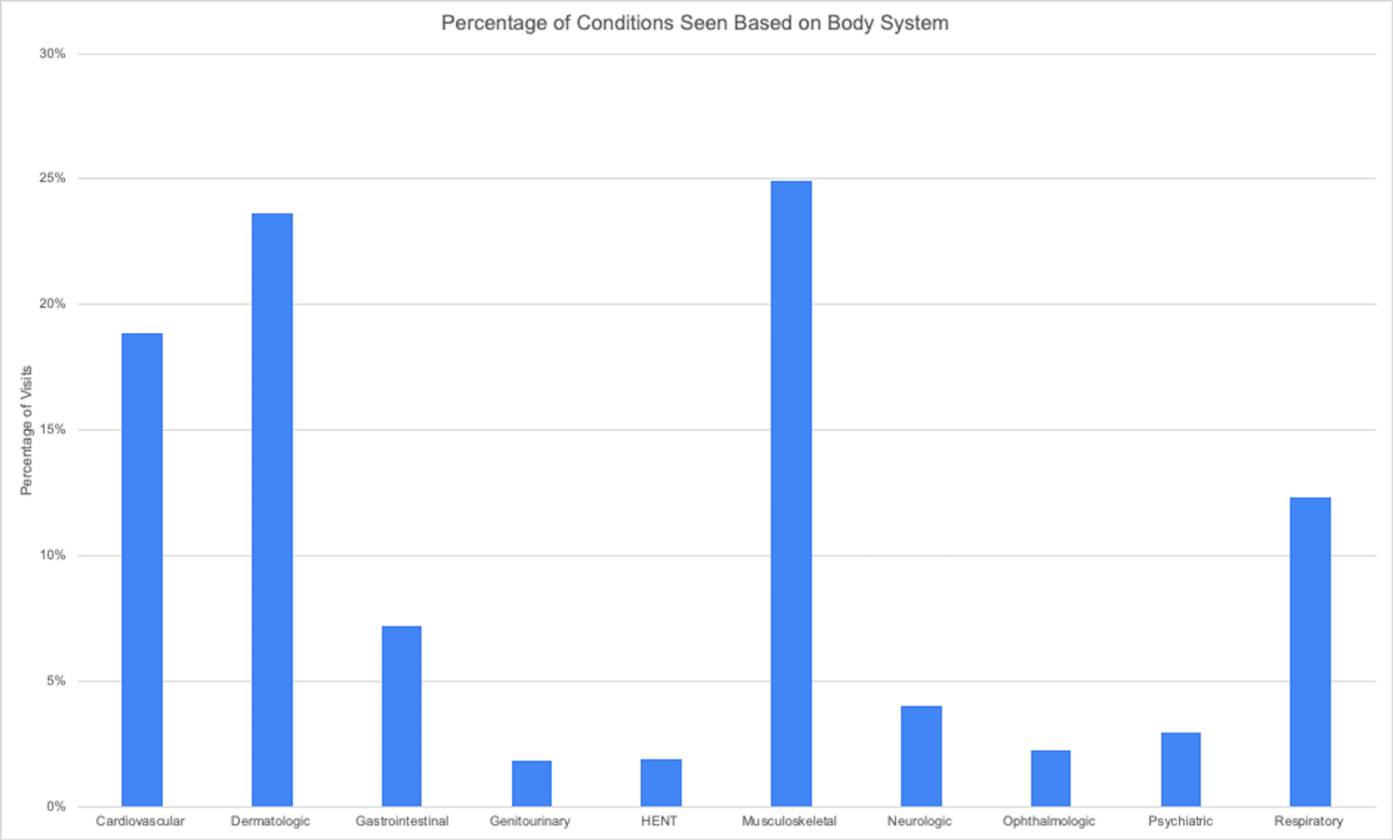
Percentage of conditions seen based on the body system at the clinic. HENT: head, ears, nose, throat

According to a retrospective internal chart review, clients presented with a variety of medical conditions across different body systems, as shown in Figure 1. The most common concerns by system were musculoskeletal (25%), dermatologic (23.7%), cardiovascular (18.9%), and respiratory (12.4%), exemplifying the wide range of conditions students learn about at the clinic.

Results of Medical Student Surveys

**Figure 2:**
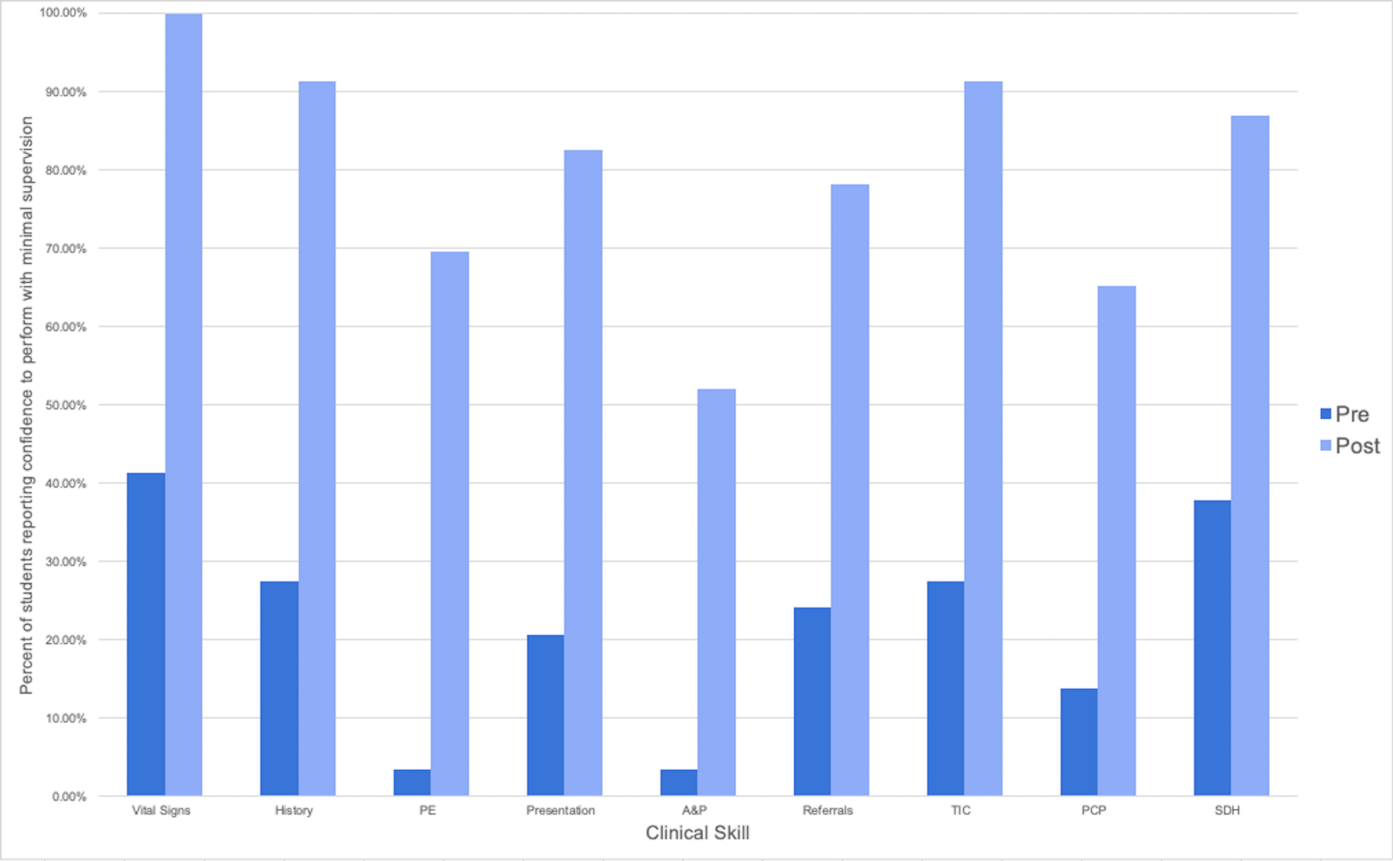
Percentage of SRFC students stating confidence in clinical skill ability in pre- vs. post-test. PE: physical examination; A&P: assessment and presentation; TIC: trauma-informed care; PCP: primary care provider; SDH: social determinants of health; SRFC: student-run free clinic

A total of 29 first-year medical students completed the pre-test survey, and 23 students completed the post-test survey. This is a response rate of 96.6% for the pre-test and 76.6% for the post-test survey. All of the questions asked showed a statistically significant increase in confidence when comparing the pre- and post-survey responses (Table 1). Figure 2 shows the percentages of students who identified themselves as comfortable performing the skill independently with minimal, passive supervision in the pre- versus post-test. Additionally, the majority of students (19 out of 23) stated that they felt like their time at the SRFC provided experience that sufficiently prepared them for clinical rotations. Three responded that they found improvement in their skills, but did not yet feel fully prepared for rotations.

**Table 1:** Pre- and post-survey results from medical students. MCP: Mobile Clinic Project

Question: How comfortable are you in performing the following activity	P-value comparing pre- vs. post-survey responses
Taking accurate vital signs	<0.00001
Obtaining an accurate and focused medical history (including history of present illness, relevant past medical and social history, and current medication list)	<0.00001
Conducting a focused physical examination	<0.00001
Summarizing the medical history into a concise presentation to the attending physician	<0.00001
Beginning to generate an assessment, differential diagnosis, and management plan	0.0001
Interacting with different members of the MCP care team to connect clients to medical referrals and social services	0.0002
Conducting a medical interview using trauma-informed language and examination skills	<0.00001
Counseling a client on referrals to primary, longitudinal medical care	0.0002
Determining the social determinants of health and how they affect our clients at MCP	0.0005

Qualitative findings

Three overarching themes were identified among the open-ended responses from participants that also aligned with the purpose of integrating an SRFC into the medical school curriculum.

Theme: Preparation for Clinical Rotations With Real-Life Applications

The EACE experience is often the first clinical experience that students have while in medical school. Students felt as though they were able to apply what they learned in the classroom to real patients:

“[The SRFC] has given me a lot of opportunity to apply the doctoring skill(s) we learn in [the classroom] in real-life situations ... it has been excellent preparation for clinical rotations.”

“...[I am able to] continue practicing my oral presentations and practice putting together a plan to be subsequently presented. I feel much more prepared for clinical rotations due to the practical nature of the [SRFC].”

Other students commented on the ability to work with different supervising attendings, which “[offered] an amazing opportunity to hone my skills after feedback from multiple physicians. I am now well acquainted with conducting solo visits, targeted physical exams, coming up with [an] A&P, and presenting to the attending physician - all crucial skills for clerkships.”

Another student stated that it should be “required for all students as it gives a good comprehensive experience on what it would be like on rotations.”

Despite this positive feedback, several students expressed inadequate preparation for their core rotations and noted limitations in their learning experience related to the environment of the student-run clinic.

“[The SRFC] offers beneficial, direct, and mostly unsupervised experiences with patient care, but by its nature, it does not fully prepare us for the hospital environment.”

“While I still feel unprepared for rotations, due to the work being done at [the SRFC], it is much less so than had I been at other sites.”

“I feel prepared to gather [a] history and present [to an attending]. I don't feel prepared for physical exams, as I did mostly skin exams.”

Although SRFCs appear to provide numerous benefits, it is clear that SRFCs do not provide a comprehensive real-life application of clinical skills. As such, medical schools should continue to provide early clinical experiences in a variety of settings.

Theme: Independence as a Medical Student Within a Safe Learning Space

Given the structure of our clinic, medical students found themselves with a unique opportunity to work independently with their patients, commenting:

“I felt like I was treated like an actual resident in the fact that I was allowed to do everything, including taking the history and physical and creating my differential and plan.”

This independence allowed students to feel more certainty in their abilities as providers.

“[The SRFC] allows you to begin to feel more confident about obtaining/performing all of the above features, which for sure prepare us for clerkships.”

“The confidence in having done the whole clinical process during [the SRFC] feels invaluable.”

Furthermore, despite the complexity of patients and the unique setting, students still felt well supported.

“I was able to practice basic clinical skills such as oral presentations, physical exam, [and] organizing a differential diagnosis all in a very low-stakes and encouraging setting.”

“I most enjoyed … the ability to take full histories without much time constraint, and the ability to ask attendings pertinent questions while we worked through a [different diagnosis] and [assessment and plan].”

Theme: Exposure to Street-Side Medicine and Humanism

Having exposure to a street-side clinic and working with unhoused patients also offered the opportunity to practice humanism.

“...[The] combination of having clients that I care about and want to do best by as well as a fantastic team to learn from immersed me in street medicine better than anything else I had done.”

“...[The SRFC] taught me not only clinical skills but also life skills/lessons given that we were working with such a unique population of patients.”

“I most enjoyed the continuity of seeing the same patients week after week.”

## Discussion

This mixed-methods study highlights the utility of integrating SRFCs into the preclinical medical school curriculum. Our findings indicate that SRFCs offer exposure to a range of medical conditions, opportunities to hone clinical skills, and a chance to build team-based and humanistic approaches to care in early medical student learners.

Broad disease pathology exposure

First, our chart review shows that our SRFC sees a broad range of medical conditions spanning different organ systems. This allows early medical learners the chance to see varied disease pathologies and acquire medical knowledge across a broad range of conditions. While certain types of concerns are more common than others, particularly in the unhoused population, this also allows students to see the effects of the SDHs firsthand. The breadth of disease seen at an SRFC may contribute to, be worsened by, or be caused by being unhoused [6]. Furthermore, other studies have shown that SRFC patients have a higher likelihood of having multiple diagnoses, which allows medical students to be exposed to patients with numerous concerns as they will encounter in future practice [5].

Clinical skill development

Second, our study demonstrated that students were significantly more comfortable in their ability to perform basic clinical skills after their SRFC participation. These skills included the main tasks students would be expected to perform in early medical clerkships. Our findings were reinforced upon review of the qualitative analysis, with the majority of students endorsing clinical rotation preparedness after their SRFC experience and a number citing increased comfort with clinical tasks. While these skills are expected to be gradually acquired throughout students’ medical training, early preparation through SRFC participation may benefit students as they begin clerkships. Previous studies have also shown that greater medical student preparedness correlates with a stronger sense of self as a healthcare provider and subsequent integration into the medical team [7].

Despite significant gains after SRFC exposure, our study still found gaps in student confidence to enter clerkships, such as physical examination maneuvers. This is most likely attributable to our clinic’s location outside on the street, which often results in over-the-clothing examinations to prioritize patient privacy and comfort. We agree that early SRFC exposure will not guarantee readiness for clerkships, and practicing clinical skills in a variety of settings would be beneficial for students. Regardless, early exposure at an SRFC allows early medical students the opportunity to practice and gain confidence in a variety of clinical skills.

One of the primary limitations of this study is the lack of a control group (students who participated in a different early clinical experience site that was not an SRFC), as the medical school declined to let us survey students outside our SRFC course site. Other studies examining early, non-SRFC, clinical experiences with direct patient involvement have shown similar findings regarding overall positive benefits for learners in terms of their clinical skills [8,9]. As a result, it is unclear if the increase in confidence in clinical skill is unique to the students’ experience at the SRFC or rather a culmination of the acquired clinical skills throughout the first year of medical school. However, a recent study of second to fourth-year medical students, who were more advanced than our surveyed first years, also found increased confidence in history taking, physical examination, teamwork, and clerkship preparedness with SRFC participation [4]. Additionally, several of the qualitative responses in our study suggest that the SRFC experience may be a significant contributing factor. Students specifically commented on the supportive independence and responsibilities afforded to medical students in SRFCs and how having this autonomy allowed for confidence entering clinical rotations, a finding similar to other studies of early clinical experiences [10].

Interdisciplinary collaboration

While exposure at many clinical sites would allow early medical students the opportunity to practice clinical skills, working at an SRFC uniquely exposes students to other aspects of healthcare. This includes practice working with an interdisciplinary team, a skill our students felt more confident with by the end of their SRFC experience. This interdisciplinary exposure leads to increased understanding of other health professionals and improved attitudes toward interprofessional teamwork [11-13].

Additionally, at an SRFC, students can appreciate the need for primary longitudinal medical care as they see the challenges of managing chronic conditions, addressing preventative health, or navigating the healthcare system without a primary care physician. As our study showed, students felt more comfortable referring patients to primary care after their work at our SRFC. This is important as increased use of primary care will help decrease healthcare utilization costs and improve continuity of care for this vulnerable population [14,15].

Working with vulnerable communities

Beyond a systems-based understanding of healthcare, our survey also shows how early clinical exposure at an SRFC affords students the opportunity to build confidence in understanding the SDHs and delivering trauma-informed care. At SRFCs, students witness firsthand how economic forces, social factors, mental health, substance use, and systemic challenges affect our unhoused patients every day and the impact this has on managing their health [16,17]. As a result of this exposure, students gain skills in dealing with these challenges, in particular, by practicing trauma-informed care. This is particularly important given the high rates of trauma and adverse childhood experiences in the United States and the fact that trauma-informed care has been shown to improve mental health, increase trauma disclosure, and decrease healthcare costs [18-22].

Finally, although not definitively delineated through the survey, qualitative responses revealed a third theme of students seeing their SRHC patients as teachers and connecting to them on a human level, which has also been noted in other studies [5]. Though this was not a primary focus of our study, it is worth noting that another benefit of having SRFC participation integrated into the medical curriculum is increased empathy and positive reinforcement toward working with the underserved [23,24].

Limitations

Other limitations of this study include a small sample size, which limits our statistical power. Though we had a high response rate, only 15 students per year were enrolled in our SRFC site. Our single location at the University of California, Los Angeles may also limit generalizability to other SRFCs, given variability in how these clinics are structured and run. Nonetheless, we feel the findings of this study showing improvement in first-year medical student learners’ clinical skills after participating in an SRFC site integrated into the medical school curriculum are notable and worthy of further research in other medical school curricula.

## Conclusions

SRFCs are an avenue for PEH to access care and are often integrated into medical schools, though not as part of the formal curriculum. Minimal research exists showing how early medical student training in an SRFC contributes to the development of crucial clinical skills and readiness for clinical clerkships. Our study has shown that students gained confidence and comfort in a multitude of tasks, taking into account the possible confounding factors, and strengthened the toolkit of students entering their clinical rotations. Working in an SRFC improved engagement in clinical care, perceived clinical preparedness, and relationships with vulnerable communities. Future research endeavors should explore the differences in comfort between participation in an SRFC and other early clinical experiences to understand its unique impact and consider reassessing students later in medical school to better understand the impact of SRFC participation in clerkship rotations.

## Figures and Tables

**Figure 3:**
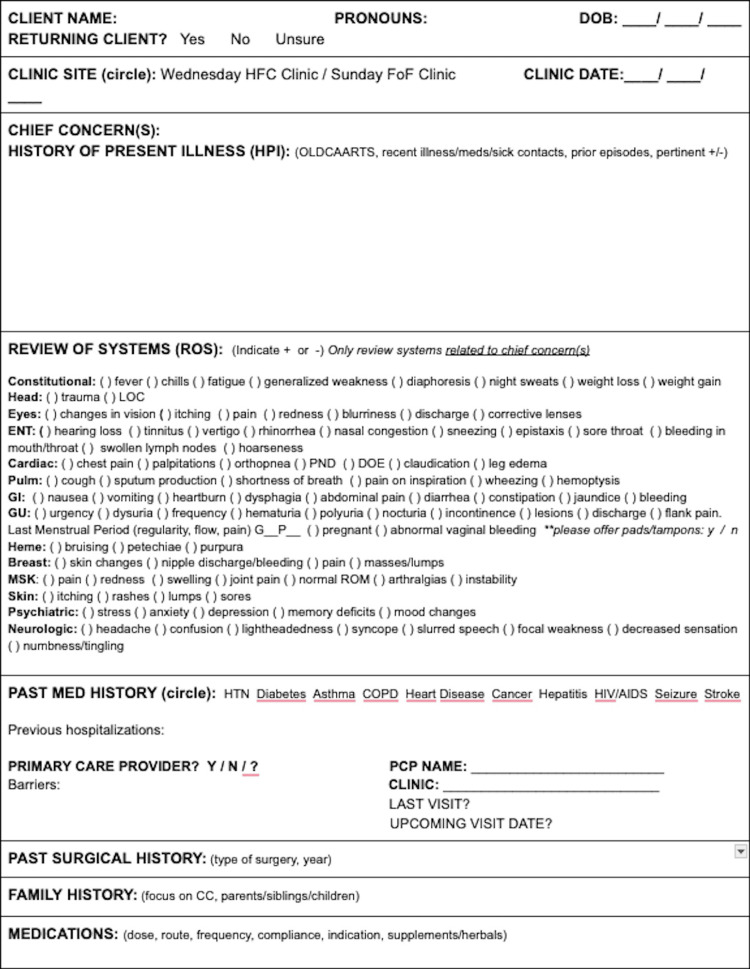
Medical history template page 1.

**Figure 4:**
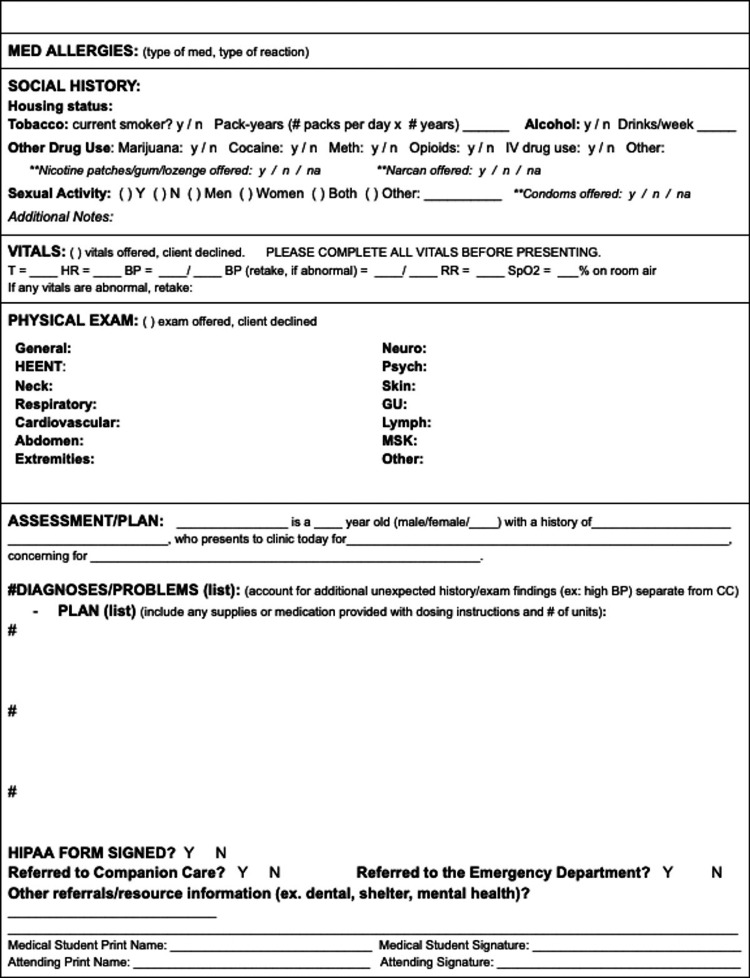
Medical history template page 2.

## Appendices

 Appendix A

Appendix B

Pre-survey and post-survey questions:

How comfortable are you in performing the following activity:

1.      Taking accurate vital signs

I can perform independently with minimal, passive supervision

I can perform, but need active attending support

I need significant guidance and support from the attending

This is an area of concern for me, and I would like to speak with a faculty member

How comfortable are you in performing the following activity:

2.     Obtaining an accurate and focused medical history, including history of present illness, relevant past medical and social history, and current medication list.

I can perform independently with minimal, passive supervision

I can perform, but need active attending support

I need significant guidance and support from the attending

This is an area of concern for me, and I would like to speak with a faculty member

How comfortable are you in performing the following activity:

3.     Conducting a focused physical exam.

I can perform independently with minimal, passive supervision

I can perform, but need active attending support

I need significant guidance and support from the attending

This is an area of concern for me, and I would like to speak with a faculty member

How comfortable are you in performing the following activity:

4.     Summarizing the medical history into a concise presentation to the attending physician

I can perform independently with minimal, passive supervision

I can perform, but need active attending support

I need significant guidance and support from the attending

This is an area of concern for me, and I would like to speak with a faculty member

How comfortable are you in performing the following activity:

5.     Beginning to generate an assessment, differential diagnosis, and management plan

I can perform independently with minimal, passive supervision

I can perform, but need active attending support

I need significant guidance and support from the attending

This is an area of concern for me, and I would like to speak with a faculty member

How comfortable are you in performing the following activity:

6.      Interacting with different members of the MCP care team to connect clients to medical referrals and social services

I can perform independently with minimal, passive supervision

I can perform, but need active attending support

I need significant guidance and support from the attending

This is an area of concern for me, and I would like to speak with a faculty member

How comfortable are you in performing the following activity:

7.    Conducting a medical interview using trauma-informed language and examination skills

I can perform independently with minimal, passive supervision

I can perform, but need active attending support

I need significant guidance and support from the attending

This is an area of concern for me, and I would like to speak with a faculty member

How comfortable are you in performing the following activity:

8.     Counseling a client on referrals to primary, longitudinal medical care

I can perform independently with minimal, passive supervision

I can perform, but need active attending support

I need significant guidance and support from the attending

This is an area of concern for me, and I would like to speak with a faculty member

How comfortable are you in performing the following activity:

9.  Describing the social determinants of health and how they affect our clients at MCP (including but not limited to race, gender, sexual orientation, economic forces, housing status, food insecurity, language, immigration status, and access to both medical care and mental health care).

I can perform independently with minimal, passive supervision

I can perform, but need active attending support

I need significant guidance and support from the attending

This is an area of concern for me, and I would like to speak with a faculty member

Appendix C

Post-survey additional open-ended questions

Did you think the MCP EACE experience allowed you to acquire and practice clinical skills to be sufficiently prepared for clinical rotations? Why or why not?

Is there anything you would change about the MCP EACE for the future?

Please provide any additional feedback, questions, or concerns about your experience at MCP or opportunities for MCP EACE improvement.
